# 1-Benzyl-1,4-diazepan-5-one

**DOI:** 10.1107/S1600536808001773

**Published:** 2008-02-08

**Authors:** Fei Sha, Hao Xu, Shan Liu, Peng Wang, Jin-tang Wang

**Affiliations:** a Department of Applied Chemistry, College of Sciences, Nanjing University of Technology, Xinmofan Road No. 5 Nanjing, Nanjing 210009, People’s Republic of China; b Center of Drug Discovery, China Pharmaceutical University, Nanjing 210009, People’s Republic of China

## Abstract

The title compound, C_12_H_16_N_2_O, is a diazepane inter­mediate that can be used as an inhibitor of human nitric oxide synthesis. In the mol­ecule, the seven-membered ring has a chair-like conformation and the two rings are approximately perpendicular to one another, with a C—N—C—C torsion angle of 77.8 (4)°. Inter­molecular N—H⋯O hydrogen bonds link the mol­ecules into dimers around a centre of symmetry, with C—H⋯O inter­actions linking the dimers into infinite sheets.

## Related literature

For related literature, see: Gopalakrishnan *et al.* (2007 ▶); Wlodarczyk *et al.* (2006 ▶).
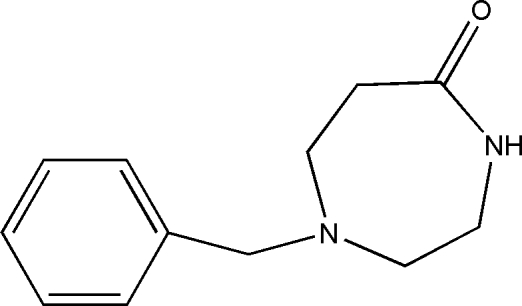


## Experimental

### 

#### Crystal data

C_12_H_16_N_2_O
*M* *_r_* = 204.27
Monoclinic, 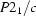

*a* = 12.602 (3) Å
*b* = 7.4920 (15) Å
*c* = 12.824 (3) Å
β = 111.00 (3)°
*V* = 1130.3 (4) Å^3^
*Z* = 4
Mo *K*α radiation
μ = 0.08 mm^−1^
*T* = 298 (2) K
0.20 × 0.10 × 0.10 mm

#### Data collection

Enraf–Nonius CAD-4 diffractometer
Absorption correction: ψ scan (North *et al.*, 1968 ▶) *T*_min_ = 0.985, *T*_max_ = 0.992
2308 measured reflections
2205 independent reflections
1162 reflections with *I* > 2σ(*I*)
*R*_int_ = 0.053
3 standard reflections every 200 reflections intensity decay: none

#### Refinement

*R*[*F* ^2^ > 2σ(*F* ^2^)] = 0.088
*wR*(*F* ^2^) = 0.189
*S* = 0.99
2205 reflections
130 parameters
46 restraints
H-atom parameters constrained
Δρ_max_ = 0.52 e Å^−3^
Δρ_min_ = −0.18 e Å^−3^

### 

Data collection: *CAD-4 Software* (Enraf–Nonius, 1989 ▶); cell refinement: *CAD-4 Software*; data reduction: *XCAD4* (Harms & Wocadlo, 1995 ▶); program(s) used to solve structure: *SHELXS97* (Sheldrick, 2008 ▶); program(s) used to refine structure: *SHELXL97* (Sheldrick, 2008 ▶); molecular graphics: *SHELXTL* (Sheldrick, 2008 ▶); software used to prepare material for publication: *SHELXTL*.

## Supplementary Material

Crystal structure: contains datablocks global, I. DOI: 10.1107/S1600536808001773/fl2179sup1.cif

Structure factors: contains datablocks I. DOI: 10.1107/S1600536808001773/fl2179Isup2.hkl

Additional supplementary materials:  crystallographic information; 3D view; checkCIF report

## Figures and Tables

**Table 1:** Hydrogen-bond geometry (Å, °)

*D*—H⋯*A*	*D*—H	H⋯*A*	*D*⋯*A*	*D*—H⋯*A*
N1—H1*A*⋯O^i^	0.86	2.00	2.821 (5)	160
C4—H4*B*⋯O^ii^	0.97	2.51	3.377 (5)	149

Symmetry codes: (i) 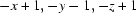; (ii) 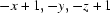.

**Fig. 1.**
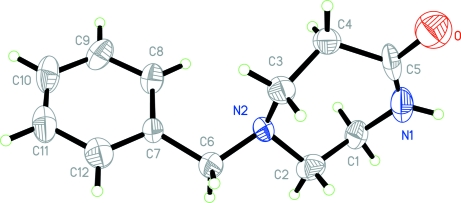
The molecular structure of the title molecule, with the atom-numbering scheme. Displacement ellipsoids are drawn at the 30% probability level.

**Fig. 2.**
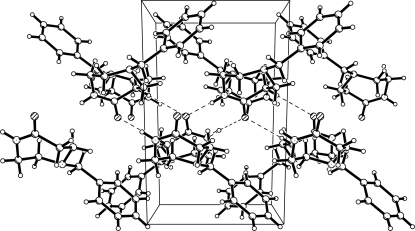
A packing diagram for (I). Hydrogen bonds are shown as dashed lines.

## supplementary crystallographic information

### Comment

The title compound is a 1-substituted 1,4-diazepan-5-one; an important class of heterocyclic compounds that have widespread applications from pharmaceuticals (Wlodarczyk *et al.*, 2006) to biology (Gopalakrishnan *et al.*, 2007). As part of our studies in this area, we report herein the synthesis and crystal structure of the title compound, (I).

In the molecule (Fig. 1) the 7-membered ring has a chair-like conformation with C1,C2,C3 and C4 forming the planar seat of the chair and N2 out of the plane on one side and N1—C5 out of the plane on the other side. The two rings are approximately perpendicular to one another with a C3—N2—C6—C7 torsion angle of 77.8 (4)°. Intermolecular N—H···O hydrogen bonds link the molecules into dimers around a center of symmetry with C—H···O interactions linking the dimers into infinite sheets (Fig. 2).

### Experimental

1-Benzyl-piperidin-4-one (18.9 g,0.1 mol) was added into a stirred mixture of sulfuric acid (40 ml) and dichloromethane (80 ml) at 273 K. Then, at 273 K, sodium azide (32.5 g,0.5 mol) was cautiously added over a period of 3 h and the resulting mixture was stirred for 1 h with the temperature kept at approximately 278 K. Then ice (1 kg) was quickly added and the solution was alkalized with ammonium hydroxide (15%,200 mL) to pH=11. The organic layer was separated with the water fraction extracted with dichloromethane (3x100mL). The organic extracts were combined,dried over NaSO_4_, and concentrated *in vacuo*. The residue was recrystallized from EtOAc to give the title compound, (I) (yield: 13.0 g, 65%). Crystals of (I) suitable for X-ray analysis were obtained by slow evaporation of an ethanol solution.

### Refinement

H atoms were positioned geometrically, with N—H = 0.86 Å (for NH) and 0.93 Å fro aromatic carbons and 0.97 Å for all others, and constrained to ride on their parent atoms, with *U*_iso_(H) = xUeq(C,*N*), where *x* = 1.5 for methyl H, and *x* = 1.2 for all other H atoms.

### Crystal data

**Table:**

C_12_H_16_N_2_O	*F*_000_ = 440
*M**_r_* = 204.27	*D*_x_ = 1.200 Mg m^−^^3^
Monoclinic, *P*2_1_/*c*	Mo *K*α radiation λ = 0.71073 Å
Hall symbol: -P 2ybc	Cell parameters from 25 reflections
*a* = 12.602 (3) Å	θ = 9–12º
*b* = 7.4920 (15) Å	µ = 0.08 mm^−^^1^
*c* = 12.824 (3) Å	*T* = 298 (2) K
β = 111.00 (3)º	BLOCK, colourless
*V* = 1130.3 (4) Å^3^	0.20 × 0.10 × 0.10 mm
*Z* = 4	

### Data collection

**Table:**

Enraf–Nonius CAD-4 diffractometer	*R*_int_ = 0.053
Radiation source: fine-focus sealed tube	θ_max_ = 26.0º
Monochromator: graphite	θ_min_ = 1.7º
*T* = 298(2) K	*h* = −15→14
ω/2θ scans	*k* = 0→9
Absorption correction: ψ scan(North *et al.*, 1968)	*l* = 0→15
*T*_min_ = 0.985, *T*_max_ = 0.992	3 standard reflections
2308 measured reflections	every 200 reflections
2205 independent reflections	intensity decay: none
1162 reflections with *I* > 2σ(*I*)	

### Refinement

**Table:**

Refinement on *F*^2^	Secondary atom site location: difference Fourier map
Least-squares matrix: full	Hydrogen site location: inferred from neighbouring sites
*R*[*F*^2^ > 2σ(*F*^2^)] = 0.088	H-atom parameters constrained
*wR*(*F*^2^) = 0.189	*w* = 1/[σ^2^(*F*_o_^2^) + (0.04*P*)^2^ + 1.65*P*] where *P* = (*F*_o_^2^ + 2*F*_c_^2^)/3
*S* = 1.00	(Δ/σ)_max_ < 0.001
2205 reflections	Δρ_max_ = 0.52 e Å^−^^3^
130 parameters	Δρ_min_ = −0.18 e Å^−^^3^
46 restraints	Extinction correction: none
Primary atom site location: structure-invariant direct methods	

### Special details

**Table:**

Geometry. All e.s.d.'s (except the e.s.d. in the dihedral angle between two l.s. planes) are estimated using the full covariance matrix. The cell e.s.d.'s are taken into account individually in the estimation of e.s.d.'s in distances, angles and torsion angles; correlations between e.s.d.'s in cell parameters are only used when they are defined by crystal symmetry. An approximate (isotropic) treatment of cell e.s.d.'s is used for estimating e.s.d.'s involving l.s. planes.
Refinement. Refinement of *F*^2^ against ALL reflections. The weighted *R*-factor *wR* and goodness of fit *S* are based on *F*^2^, conventional *R*-factors *R* are based on *F*, with *F* set to zero for negative *F*^2^. The threshold expression of *F*^2^ > σ(*F*^2^) is used only for calculating *R*-factors(gt) *etc*. and is not relevant to the choice of reflections for refinement. *R*-factors based on *F*^2^ are statistically about twice as large as those based on *F*, and *R*- factors based on ALL data will be even larger.

### Fractional atomic coordinates and isotropic or equivalent isotropic displacement parameters (Å^2^)

**Table:**

	*x*	*y*	*z*	*U*_iso_*/*U*_eq_	
N1	0.3935 (3)	−0.4319 (6)	0.5667 (3)	0.0870 (12)	
H1A	0.4312	−0.5275	0.5667	0.104*	
O	0.5027 (3)	−0.2681 (5)	0.4894 (3)	0.108	
N2	0.2757 (3)	−0.1384 (4)	0.6724 (2)	0.0542 (8)	
C1	0.2993 (4)	−0.4434 (5)	0.6094 (4)	0.0705 (12)	
H1B	0.2940	−0.5657	0.6319	0.085*	
H1C	0.2289	−0.4158	0.5487	0.085*	
C2	0.3090 (4)	−0.3232 (5)	0.7059 (4)	0.0697 (11)	
H2A	0.2613	−0.3696	0.7446	0.084*	
H2B	0.3869	−0.3245	0.7579	0.084*	
C3	0.3581 (3)	−0.0442 (5)	0.6349 (3)	0.0632 (10)	
H3A	0.4334	−0.0571	0.6912	0.076*	
H3B	0.3396	0.0820	0.6278	0.076*	
C4	0.3596 (3)	−0.1132 (6)	0.5236 (3)	0.0683 (11)	
H4A	0.2817	−0.1299	0.4735	0.082*	
H4B	0.3932	−0.0222	0.4913	0.082*	
C5	0.4230 (3)	−0.2848 (7)	0.5289 (4)	0.0765 (13)	
C6	0.2595 (3)	−0.0404 (6)	0.7642 (3)	0.0685 (12)	
H6A	0.3332	−0.0018	0.8157	0.082*	
H6B	0.2272	−0.1204	0.8043	0.082*	
C7	0.1827 (3)	0.1221 (5)	0.7265 (3)	0.0531 (9)	
C8	0.1029 (3)	0.1333 (5)	0.6142 (4)	0.0643 (11)	
H8A	0.1017	0.0489	0.5606	0.077*	
C9	0.0285 (4)	0.2739 (6)	0.5892 (4)	0.0779 (13)	
H9A	−0.0223	0.2845	0.5161	0.094*	
C10	0.0241 (4)	0.3960 (7)	0.6626 (5)	0.0865 (15)	
H10A	−0.0288	0.4879	0.6406	0.104*	
C11	0.0969 (5)	0.3858 (6)	0.7691 (5)	0.0869 (15)	
H11A	0.0954	0.4710	0.8213	0.104*	
C12	0.1748 (4)	0.2447 (6)	0.7999 (4)	0.0713 (12)	
H12A	0.2230	0.2354	0.8741	0.086*	

### Atomic displacement parameters (Å^2^)

**Table:**

	*U*^11^	*U*^22^	*U*^33^	*U*^12^	*U*^13^	*U*^23^
N1	0.065 (2)	0.109 (3)	0.076 (3)	0.031 (2)	0.0121 (19)	−0.027 (2)
O	0.108	0.108	0.108	0.000	0.039	0.000
N2	0.0666 (19)	0.0520 (18)	0.0461 (17)	0.0091 (16)	0.0229 (15)	−0.0087 (15)
C1	0.084 (3)	0.048 (2)	0.083 (3)	0.011 (2)	0.034 (2)	−0.003 (2)
C2	0.085 (3)	0.050 (2)	0.073 (3)	−0.004 (2)	0.027 (2)	0.011 (2)
C3	0.072 (3)	0.055 (2)	0.070 (3)	−0.005 (2)	0.035 (2)	0.002 (2)
C4	0.054 (2)	0.089 (3)	0.065 (2)	0.012 (2)	0.0255 (19)	0.016 (2)
C5	0.051 (2)	0.100 (3)	0.084 (3)	0.017 (2)	0.030 (2)	−0.033 (3)
C6	0.069 (3)	0.079 (3)	0.055 (2)	0.025 (2)	0.020 (2)	0.003 (2)
C7	0.060 (2)	0.048 (2)	0.058 (2)	0.0113 (18)	0.0293 (19)	0.0002 (18)
C8	0.052 (2)	0.058 (2)	0.080 (3)	0.003 (2)	0.020 (2)	−0.009 (2)
C9	0.064 (3)	0.064 (3)	0.092 (3)	0.012 (2)	0.011 (2)	0.007 (3)
C10	0.066 (3)	0.074 (3)	0.131 (5)	0.018 (3)	0.050 (3)	−0.009 (3)
C11	0.098 (4)	0.061 (3)	0.119 (4)	0.001 (3)	0.061 (4)	−0.018 (3)
C12	0.078 (3)	0.064 (3)	0.084 (3)	−0.011 (2)	0.043 (3)	−0.003 (2)

### Geometric parameters (Å, °)

**Table:**

N1—C5	1.311 (6)	C4—H4A	0.9700
N1—C1	1.477 (5)	C4—H4B	0.9700
N1—H1A	0.8600	C6—C7	1.523 (5)
O—C5	1.284 (5)	C6—H6A	0.9700
N2—C6	1.462 (4)	C6—H6B	0.9700
N2—C2	1.465 (5)	C7—C12	1.345 (5)
N2—C3	1.471 (4)	C7—C8	1.433 (5)
C1—C2	1.500 (5)	C8—C9	1.370 (5)
C1—H1B	0.9700	C8—H8A	0.9300
C1—H1C	0.9700	C9—C10	1.328 (6)
C2—H2A	0.9700	C9—H9A	0.9300
C2—H2B	0.9700	C10—C11	1.347 (7)
C3—C4	1.525 (5)	C10—H10A	0.9300
C3—H3A	0.9700	C11—C12	1.400 (6)
C3—H3B	0.9700	C11—H11A	0.9300
C4—C5	1.502 (6)	C12—H12A	0.9300
			
C5—N1—C1	124.0 (4)	H4A—C4—H4B	107.4
C5—N1—H1A	118.0	O—C5—N1	126.5 (4)
C1—N1—H1A	118.0	O—C5—C4	112.2 (5)
C6—N2—C2	110.3 (3)	N1—C5—C4	121.3 (3)
C6—N2—C3	109.9 (3)	N2—C6—C7	113.7 (3)
C2—N2—C3	112.8 (3)	N2—C6—H6A	108.8
N1—C1—C2	115.6 (4)	C7—C6—H6A	108.8
N1—C1—H1B	108.4	N2—C6—H6B	108.8
C2—C1—H1B	108.4	C7—C6—H6B	108.8
N1—C1—H1C	108.4	H6A—C6—H6B	107.7
C2—C1—H1C	108.4	C12—C7—C8	117.6 (4)
H1B—C1—H1C	107.4	C12—C7—C6	121.5 (4)
N2—C2—C1	113.3 (3)	C8—C7—C6	120.2 (3)
N2—C2—H2A	108.9	C9—C8—C7	117.1 (4)
C1—C2—H2A	108.9	C9—C8—H8A	121.5
N2—C2—H2B	108.9	C7—C8—H8A	121.5
C1—C2—H2B	108.9	C10—C9—C8	124.2 (5)
H2A—C2—H2B	107.7	C10—C9—H9A	117.9
N2—C3—C4	113.0 (3)	C8—C9—H9A	117.9
N2—C3—H3A	109.0	C9—C10—C11	119.6 (5)
C4—C3—H3A	109.0	C9—C10—H10A	120.2
N2—C3—H3B	109.0	C11—C10—H10A	120.2
C4—C3—H3B	109.0	C10—C11—C12	118.9 (5)
H3A—C3—H3B	107.8	C10—C11—H11A	120.6
C5—C4—C3	115.7 (3)	C12—C11—H11A	120.6
C5—C4—H4A	108.4	C7—C12—C11	122.6 (4)
C3—C4—H4A	108.4	C7—C12—H12A	118.7
C5—C4—H4B	108.4	C11—C12—H12A	118.7
C3—C4—H4B	108.4		
			
C5—N1—C1—C2	−58.5 (6)	C3—N2—C6—C7	77.8 (4)
C6—N2—C2—C1	165.8 (3)	N2—C6—C7—C12	−167.9 (4)
C3—N2—C2—C1	−70.9 (4)	N2—C6—C7—C8	22.3 (5)
N1—C1—C2—N2	79.5 (5)	C12—C7—C8—C9	3.2 (6)
C6—N2—C3—C4	−166.9 (3)	C6—C7—C8—C9	173.4 (4)
C2—N2—C3—C4	69.6 (4)	C7—C8—C9—C10	−1.8 (7)
N2—C3—C4—C5	−78.3 (4)	C8—C9—C10—C11	0.5 (8)
C1—N1—C5—O	−178.8 (4)	C9—C10—C11—C12	−0.7 (7)
C1—N1—C5—C4	−1.4 (7)	C8—C7—C12—C11	−3.6 (6)
C3—C4—C5—O	−122.4 (4)	C6—C7—C12—C11	−173.7 (4)
C3—C4—C5—N1	59.8 (6)	C10—C11—C12—C7	2.4 (7)
C2—N2—C6—C7	−157.2 (3)		

### Hydrogen-bond geometry (Å, °)

**Table:**

*D*—H···*A*	*D*—H	H···*A*	*D*···*A*	*D*—H···*A*
N1—H1A···O^i^	0.86	2.00	2.821 (5)	160
C4—H4B···O^ii^	0.97	2.51	3.377 (5)	149

Symmetry codes: (i) −*x*+1, −*y*−1, −*z*+1; (ii) −*x*+1, −*y*, −*z*+1.
